# Psychopathology and Other Mental Health Challenges in Siblings of Patients with Child- or Adolescent-Onset Anorexia Nervosa: A Systematic Review with a Sex/Gender Perspective

**DOI:** 10.3390/jcm15051772

**Published:** 2026-02-26

**Authors:** Elisabet Tasa-Vinyals, Maria Teresa Plana, Albert Martínez-Pinteño, Mireia Mora-Porta, Arturo Rodríguez-Rey, Susana Andrés-Perpiñá, Elena Moreno, Esteban Martínez, Josefina Castro-Fornieles, Itziar Flamarique

**Affiliations:** 1 Department of Child and Adolescent Psychiatry and Psychology, Institut Clínic de Neurociènces, Hospital Clinic of Barcelona, 08036 Barcelona, Spain; 2 Fundació de Recerca Clínic Barcelona-Institut d’Investigacions Biomèdiques August Pi i Sunyer (IDIBAPS), 08036 Barcelona, Spain; 3 Faculty of Medicine, University of Barcelona, 08036 Barcelona, Spain; 4 Department of Endocrinology and Nutrition, Institut Clínic de Malalties Digestives i Metabòliques, Hospital Clinic of Barcelona, 08036 Barcelona, Spain; 5 Centro de Investigación Biomédica en Red Salud Mental—Instituto de Salud Carlos III, 28029 Madrid, Spain; 6 Faculty of Psychology, University of Barcelona, 08036 Barcelona, Spain

**Keywords:** anorexia nervosa, eating disorders, siblings, sister, brother, systematic review

## Abstract

**Background/Objectives**: Anorexia nervosa (AN) is a severe psychiatric-metabolic disorder with high morbidity and mortality, profoundly impacting family systems. Siblings of patients with AN constitute a vulnerable group, yet there is a lack of specific, quantitative evidence on their mental health status, particularly when examined from a sex/gender perspective. This review aimed to synthesize existing evidence on the prevalence of psychiatric symptoms, diagnoses, and other mental health challenges in siblings of individuals with AN and critically analyze it from a sex/gender perspective. **Methods**: Following PRISMAguidelines, a systematic search was conducted across five databases (April–May 2025). The protocol was registered in PROSPERO (CRD420251025535). Sixteen studies published between 1983 and 2022 met eligibility criteria. **Results**: Included studies were rated as high or moderate quality, and overall risk of bias was estimated as low–moderate. Only half of the studies included brothers, and overall quality assessment from a sex/gender perspective was modest. Dimensional psychopathology assessments found increased prevalence of both internalizing and externalizing symptoms in siblings compared to controls, although siblings were generally more like controls than affected probands in psychometric evaluations. The few studies based on clinically diagnosed psychopathology state that siblings are at increased risk of several mental health disorders. **Conclusions:** Specific quantitative evidence on psychiatric diagnoses, psychopathology and other mental health challenges in siblings of individuals with AN is relatively scarce and biased, particularly by systematic exclusion of externalizing symptoms and male siblings. Future translational research should be designed and interpreted using a sex/gender perspective and prioritize systematic assessment by clinicians.

## 1. Introduction

### 1.1. Anorexia Nervosa: Clinical Overview and Relevance

Anorexia nervosa (AN) is a complex and severe disorder classified under the Feeding and Eating Disorders Section in the Diagnostic and Statistical Manual of Mental Disorders, Fifth Edition (DSM-5) [1]. AN is a psychiatric-metabolic disease [2,3,4,5,6,7] characterized by:

(a) Persistent restriction of energy intake resulting in significantly low body weight or failure to achieve expected growth trajectories in children and adolescents;
(b) An intense fear of gaining weight or becoming fat, even in the context of underweight status;
(c) A distorted perception of body weight and shape, often accompanied by denial of the seriousness of the condition and its medical consequences.

Historically, a strict interpretation of criterion (a) referring to underweight condition prevented many patients presenting compatible clinical features from receiving a diagnosis of AN. This limitation was partially overcome by the introduction of atypical-presentation AN as a new diagnosis in DSM-5, where it is specifically listed and described under the epigraph of Other Specified Eating or Feeding Disorder (OSFED). According to this description, atypical AN (OSFED-AN) can be diagnosed if all criteria for AN are met except for the patient’s weight remaining within or above the normal range despite significant weight loss [1]. This diagnostic innovation of DSM-5 has significantly contributed to advancing research, clinical practice, and social awareness by underlining that individuals of all weights can have an eating disorder (ED). In addition, it has allowed many patients to receive timely, appropriate, and specific treatment for their condition. In the previous edition of DSM, OSFED-AN patients used to be classified under the umbrella term Unspecified Eating Disorder, which nonetheless included an explicit definition of atypical-presentation AN by mentioning cases where all criteria for AN are met except for underweight and amenorrhea [8]. Since amenorrhea is not a required criterion for AN diagnosis anymore, its absence has become irrelevant from DSM-5 onwards; therefore, this symptom is not mentioned in the OSFED-AN definition.

AN predominantly affects adolescent females, with a reported female-to-male ratio of up to 13:1 [9]. Its lifetime prevalence is estimated to be around 0.3–4% [9]. Importantly, AN is associated with one of the highest mortality rates among psychiatric disorders, due to both medical complications (e.g., cardiac arrhythmias, electrolyte imbalances, hematological abnormalities) and suicide [10,11]. These figures underscore the life-threatening nature of the disorder. AN can be considered the most prevalent and life-threatening of all specified EDs.

The etiopathogenesis of AN is multifactorial, involving a complex interplay of biological, psychological, and sociocultural factors. Many contributors are currently well-described in the medical literature and include genetic predisposition, neuroendocrine mechanisms, psychological functioning traits, and sociocultural influences [12]. Several pathways to disease have been described in mixed-methods narrative studies about AN [13], which are consistent with the biomedical and epidemiological data on the multifactorial origin of the disease. Current evidence supports the centrality of sex/gender characteristics and roles all along the complex processes of sickening, recovering and, in some cases, relapsing.

Comorbidity between AN and other mental health conditions is frequent, particularly obsessive–compulsive disorder, anxiety disorders, and posttraumatic stress disorder [14]. Comorbidities worsen the prognosis of AN since they are associated with anxious and depressive symptoms, hospitalization rates, and suicidal behavior [15,16]. Early detection and intervention improve response to treatment and likelihood of recovery, including early detection of risk factors [17,18]. In fact, a very recent study confirms that, similarly to the case of psychoses, identifying prodromal phases and reducing the duration of untreated illness are key to prognosis improvement in EDs [19].

Clinically, AN presents with a wide spectrum of somatic and psychological symptoms. Physical manifestations include emaciation, bradycardia, hypotension, hypothermia, lanugo, amenorrhea, osteoporosis, growth retardation, and delayed puberty [11]. Psychopathological features often encompass depressive, anxious, and obsessive–compulsive symptoms focused on controlling food intake and physical activity with the aim of reducing body weight or keeping it below age- and sex-appropriate levels, typically accompanied by poor insight or denial of illness [12].

Management of AN requires a multidisciplinary approach, integrating and synchronizing medical stabilization, nutritional rehabilitation, psychotherapeutic interventions, and psychiatric care. While no pharmacological treatment is specifically approved for AN, symptomatic management may involve low-dose antipsychotics, benzodiazepines, and selective serotonin reuptake inhibitors following weight restoration [12]. Recovery is achievable but often protracted and marked by relapses. Early intervention is associated with improved outcomes, whereas chronic cases may result in enduring functional impairment and persistent medical complications [20]. Anorexic cognitions are particularly persistent, often requiring prolonged and intense psychotherapeutic and pharmacological treatments, and have been compared to obsessive and delusional symptoms in terms of severity and functional impairment [21,22,23].

### 1.2. Psychosocial Impact of Having a Sibling with a Life-Threatening Illness

The experience of growing up with a sibling affected by a life-threatening illness can exert profound and multifaceted effects on the healthy sibling’s emotional, psychological, and social development [24,25,26,27]. These effects are arguably particularly salient during neurodevelopmental stages such as childhood and adolescence, when identity formation is still in progress and emotional regulation skills are not yet fully developed.

Empirical studies indicate that between 7% and 25% of siblings in such contexts exhibit clinically significant internalizing (e.g., anxiety, depression, withdrawal) and externalizing (e.g., aggressive, disruptive behavior) symptoms [28]. Notably, from a sex/gender perspective, in a meta-analysis of studies examining siblings of chronically ill children, boys seemed more likely to present more internalizing symptoms, while girls were more likely to exhibit externalizing behaviors—a reversal of typical prevalence patterns observed in general pediatric populations [29]. A recent study of healthy siblings has reported a greater mental health impact on girls than on boys, both in terms of internalizing and externalizing psychopathology [30].

Disruption of usual family functioning is the norm when serious disease hits a household. Healthy siblings may report feeling emotionally neglected as parental attention and resources are redirected toward the ill child, who often requires intensive and prolonged medical care. This shift can result in missed developmental opportunities, such as participation in school events, reduced quality time with family members, and increased household responsibilities. Feelings of isolation, confusion, and emotional displacement are frequently reported. These findings have consistently been reported in two recent reviews focused on siblings of individuals with EDs synthetizing dozens of studies [31,32]. While some siblings demonstrate adaptive coping and resilience over time, others may experience enduring psychological sequelae, including unresolved grief, chronic anxiety, and difficulties in forming secure interpersonal relationships. The degree of distress is often modulated by the recency of the illness onset and the availability of emotional outlets and support systems [33,34].

Significantly, despite the impact being generally and at least initially negative, some individuals report transformative outcomes, reframing their experience as a catalyst for personal growth. These siblings may develop enhanced empathy, emotional maturity, and resilience. Such positive trajectories are more likely in families that foster open communication, actively include siblings in care-related discussions, and provide access to psychosocial support services. Cultural and spiritual beliefs surrounding illness, suffering, and mortality also play a role in shaping coping responses [35,36]. These protective effects have also been described in siblings of patients with EDs in the previously mentioned reviews [31,32].

Sibling-specific interventions remain scarce in many healthcare systems even though targeted support programs such as peer support groups, individual counseling, and psychoeducational resources have demonstrated efficacy in mitigating psychological distress and promoting adaptive functioning. According to the current literature, these interventions should aim to validate the sibling’s experience, enhance emotional literacy, and empower them within the family system [37,38,39,40].

### 1.3. Sibling Relationships Within the Family System

Family Systems Theory posits that families operate through interconnected subsystems, including the parent–child, sibling, marital, and co-parenting subsystems [41]. Among these, sibling relationships are particularly enduring and influential, shaping emotional, social, and psychological development from early childhood into adulthood [42].

Siblings often serve as a child’s first peers, playing a pivotal role in the acquisition of social competence, empathy, and conflict resolution skills. Children typically spend more unsupervised time with siblings than with parents or friends, making these relationships central to their everyday emotional experiences. Due to their longevity, sibling bonds frequently outlast other familial relationships, including those with parents and extended relatives, and in some cases, even surpass the duration of spousal relationships [42].

Empirical evidence suggests that warm sibling relationships—characterized by mutual affection, support, and low levels of conflict—are associated with enhanced psychological well-being, higher self-esteem, and reduced incidence of depression and loneliness. Conversely, high-conflict sibling relationships have been linked to increased emotional distress, behavioral dysregulation, and elevated risk for mental health disorders later in life [43,44].

Bowen’s theory further emphasizes the influence of sibling position (e.g., firstborn, middle child, youngest) on personality development and familial roles [41]. Firstborns are often socialized into leadership roles and tend to exhibit greater conscientiousness. Middle children may adopt the role of mediator or peacemaker, while youngest siblings are frequently perceived as more rebellious or attention-seeking. These roles are shaped by both parental expectations and patterns of inter-sibling interaction.

Siblings can function as both protective and risk-enhancing agents. Older siblings may model prosocial behaviors and provide emotional support, while in other contexts, they may introduce younger siblings to maladaptive behaviors such as substance use. More broadly, positive sibling relationships can serve as a buffer against familial stress, whereas negative relationships may exacerbate it [45].

### 1.4. What Is a Sex/Gender Perspective and Why Is It Relevant for This Issue?

Given the predominance of AN in females and its typical onset during adolescence, affected individuals often have young and/or underage siblings, obviously including brothers and sisters. Considering the profound influence of sex/gender on health outcomes, it seems essential to examine the impact of having a sibling with AN through a sex/gender-informed lens. In this article, we use the term sex/gender following a corpomaterialist feminist approach since, in our view, it seems the most comprehensive and coherent epistemic position in medical sciences, following the prestigious gender scholar Nina Lykke [46].

A sex/gender perspective in research and medical practice entails the systematic consideration of how biological sex (male/female), inextricably entangled with socially constructed gender roles (masculinity/femininity), shape pathways to health/disease, experiences, vulnerabilities, and access to care [47]. This approach recognizes the distinct yet interrelated nature of sex and gender and their role in modulating physiological and psychological responses, including coping mechanisms. In accordance, sex/gender is known to play a central role in prevalence, as a risk/protective factor, and in treatment processes and outcomes for many mental and organic disorders [48]. Conversely, sex/gender has historically been disregarded and/or regarded in oppressive manners in the context of androcentric knowledge-production and clinical practices, leading to the phenomenon known as sex/gender bias in medicine and medical research, usually associating key knowledge gaps [47]. There is current scientific consensus around the need to explicitly adopt a sex/gender perspective as a guiding analytic lens translationally, from study design to daily clinical practice, to ensure meeting basic epistemological and ethical standards [49].

The impact of AN extends way beyond the individual, significantly affecting family dynamics and sibling relationships. Therefore, the diagnosis of a child/adolescent is usually a major stressful event for their family members, who might experience stress differently if they are young and still undergoing neurodevelopment. That being said, we must stress that emotional responses to stressful events are often gendered [50]: male siblings may externalize distress (e.g., addictive or disruptive behaviors) or internalize distress (e.g., anxiety, depression) but underreport symptoms due to norms discouraging emotional expression. Conversely, female siblings may tend to experience more internalizing forms of mental distress (e.g., anxiety, depression, body image concerns), externalize distress in qualitatively different ways from males (e.g., somatic complaints, behavioral outbursts), or assume caregiving roles traditionally associated with femininity, potentially leading to altered family system dynamics (e.g., parentification) and emotional exhaustion [51]. As previously mentioned, exceptions have been reported to this rule of thumb in studies examining siblings of chronically ill children, without a particular focus on EDs [29].

Gendered expectations within families are likely to further influence sibling experiences. Girls may be expected to provide emotional support and demonstrate nurturing behaviors, whereas boys may be encouraged to remain stoic or avoid burdening others. These expectations, that children can see modeled in the attitudes and behaviors of their mothers and fathers, shape how siblings process and articulate their experiences [50].

Moreover, exposure to a sibling with AN may differentially affect body image concerns. Even in the event of a similarly increased risk of developing an ED, such individual basal risk might intersect with sex/gender and result in female siblings being more susceptible to developing disorders linked to a drive for thinness due to societal pressures, while male siblings may be more prone to muscle dysmorphia or bigorexia. Gender norms also influence help-seeking behaviors, perceptions by healthcare professionals, and the extent to which siblings’ needs are acknowledged and responded to in clinical settings [31,32].

Incorporating a sex/gender perspective when conceptualizing and designing new research is therefore critical for identifying disparities in how sisters and brothers experience and cope with a sibling’s AN. This approach facilitates the development of tailored interventions, enhances appropriateness and effectivity of psychosocial support, and deepens understanding of the broader familial impact of EDs. Furthermore, bearing a sex/gender perspective in mind when examining existing research can facilitate identification of problematic practices, key knowledge gaps, and a more critical and nuanced interpretation of studies and research results. In clinical practice, keeping a sex/gender perspective as a guiding lens arguably leads to a more appropriate and ethical standard of care for patients and their families.

### 1.5. What Is Currently Known About Siblings of Patients with AN?

Siblings of individuals diagnosed with AN during childhood or adolescence are recognized as a vulnerable population, as they are at an increased risk for developing a range of psychological and behavioral difficulties [3,14,31]. These typically include EDs, mood and anxiety disorders, and other forms of emotional dysregulation. According to the National Human Genome Research Institute [52], an individual’s parents, siblings and offspring are first-degree relatives given that they share about half of their genetic information, on average. This genetic definition might differ from other definitions of siblinghood, e.g., legal or sociological definitions. An exceptional case is that of monozygotic twins, who share all their genetic information. Environmental factors influence genetic expression on an individual level through biomolecular pathways globally known as epigenetics [53].

First-degree relatives of patients with AN have been reported to be up to 10 times more likely to receive a diagnosis of ED in their lifetime [54]. The relatively recent literature has linked AN to autism spectrum disorder and related neurodevelopmental processes, such as central coherence, set-shifting, mentalization, and interoceptive awareness [55,56,57,58,59,60]. Several mechanisms contributing to the increased risk observed in siblings have been identified in the current literature and include:

- Emotional and psychological burden. Siblings frequently report experiencing intense emotions such as anxiety, guilt, sadness, and helplessness. In many cases, they assume informal and not always age-appropriate caregiving roles, which may lead to emotional suppression, role confusion, and chronic stress [31,32,61,62,63].
- Increased risk of exposure to patterns of disordered eating and body image preoccupation. Prolonged exposure to a sibling with AN and their symptoms can heighten sensitivity to body image, food-related behaviors, and societal beauty standards. This may result in the adoption of restricted eating patterns or other maladaptive behaviors, particularly in environments lacking protective factors [64,65,66,67,68,69].
- Genetic and epigenetic contributions. As previously mentioned, the presence of a first-degree relative with an ED significantly increases the likelihood of developing similar pathology. This is attributable to both genetic predisposition and shared environmental influences, such as family attitudes toward food, weight, and appearance. Heritability of AN is estimated amongst the highest of mental disorders, between 50 and 60% [54,70]. A classic twin study back in the 1980s established a sound empirical basis for a significant genetic contribution in AN, comparing diagnostic concordance in monozygotic (0.71) and dizygotic twins (0.1) [71]. More recently, genome-wide association (GWA) and polygenic risk score (PGS) studies support the idea that AN is a complex polygenic disease, like most mental disorders [2,5], and there is increasing interest in the role of epigenetics of AN [72].
- Perceived neglect and isolation. Healthy siblings often report feeling overlooked or emotionally sidelined as parental attention is concentrated on the ill child. This perceived neglect can foster feelings of abandonment, resentment, and difficulty accessing emotional support [31,32,62,73,74,75].

Despite these risks, some siblings report that witnessing the adverse consequences of AN serves as a deterrent against engaging in similar behaviors. Protective outcomes are more likely when siblings are provided with accurate information about the illness, included in family discussions, and supported within emotionally responsive environments [31,32,76].

Factors potentially influencing siblings’ risk of mental distress and disease are summarized in Table 1.

### 1.6. Summary of Current Knowledge Gaps Identified and Objectives of This Systematic Review

Taken together, the previously discussed findings underscore the importance of recognizing siblings not merely as passive observers but as individuals whose psychological well-being is intricately linked to the family’s experience of illness. Given the observed variability in siblings’ subjective experiences and resulting mental health outcomes, targeted interventions that address their unique needs could be key strategies for promoting resilience and preventing secondary psychopathology.

There is currently a sound theoretical framework supporting the idea that the experience of siblings of patients with AN may (a) be qualitatively distinct from that of other first-degree relatives, e.g., mothers and fathers; (b) be qualitatively distinct from that of other peers close in age to the proband, e.g., friends and classmates; (c) significantly increase their risk of suffering from several mental health challenges, symptoms and diagnoses, whether directly related or unrelated with eating behavior and body image issues; (d) sometimes result in an atypical pattern regarding sex/gender and mental health challenges, symptoms and diagnoses; and (e) make them less likely to access and benefit from early detection and treatment of such problems, therefore potentially worsening their prognosis.

We have identified two recent reviews describing in detail the experiences of siblings of patients with EDs, including AN, from a mostly qualitative point of view [31,32]. Both reviews point towards elevated levels of mental suffering and psychopathology in the siblings and agree to consider them as a particularly challenging at-risk group deserving further research and clinical attention. The phenomenological experiences revealed by the siblings in these reviews converge around a series of themes including disruptions in family relationships, negative affect, and eating-related symptoms. Though some of the reported studies include male patients, most of the analyzed data come from female siblings. There also seems to be a relative bias towards exploring and/or reporting internalizing problems, on the one hand, and eating and body image issues, on the other, compared to the broad spectrum of mental symptoms and expressions of mental distress.

Taking from this standpoint, we aim to systematically review and analyze the current evidence on the prevalence of specific mental health challenges, psychiatric symptoms and/or diagnoses in male and female siblings of patients with AN, encompassing existing quantitative data on both internalizing and externalizing problems, and with a particular interest in critically assessing existing knowledge with the analytical guidance of a sex/gender perspective.

The ultimate purpose of this review is to identify and synthesize the sort of data that, in our view, carries the largest potential to ground future endeavors in translational research aimed to understanding and improving the mental health of siblings of patients with AN.

## 2. Materials and Methods

### 2.1. Search Strategy

This systematic review was conducted in accordance with the Preferred Reporting Items for Systematic Reviews and Meta-Analyses (PRISMA, Supplementary Materials) guidelines [77]. To promote transparency and contribute to the efficient use of global research resources, the review protocol was prospectively registered in the International Prospective Register of Systematic Reviews (PROSPERO; https://www.crd.york.ac.uk/PROSPERO, accessed on 7 April 2025) under registration number CRD420251025535.

Systematic searches were performed across five major databases—PubMed (MEDLINE), PsycInfo (PsycNet), Scopus, Web of Science, and Cochrane Central—between 7 April and 29 May 2025. No restrictions were applied regarding publication date since our aim was to maximize search sensitivity. Language restrictions were applied based on the linguistic competencies of the research team.

Search terms were derived from the Medical Subject Headings (MeSH) database. “Anorexia Nervosa” is categorized under “Feeding and Eating Disorders,” which includes related conditions such as avoidant/restrictive food intake disorder, binge-eating disorder, bulimia nervosa, diabulimia, orthorexia nervosa, pica, and rumination syndrome. Broader terms such as disordered eating behavior, feeding and eating disorders of childhood, food addiction, night eating syndrome, relative energy deficiency in sport, and female athlete triad syndrome were also considered. The MeSH term “Siblings” encompasses sibling*, brother*, and sister*. The term “Sibling Relations” was excluded due to its focus on systemic constructs beyond the scope of this review.

To ensure comprehensive coverage, the final search strategy combined the terms “Feeding and Eating Disorders” AND “Siblings” across all fields.

### 2.2. Inclusion and Exclusion Criteria

Inclusion criteria:

I1: Studies involving full siblings (including twins, dizygotic and monozygotic) of individuals diagnosed with AN (typical or atypical) according to DSM or International Classification of Diseases (ICD) criteria, regardless of clinical subtype and of severity. That includes DSM-5/ICD-10 diagnostic codes F50.01/307.1 AN restricting type (ANr), F50.02/307.1 AN binge-eating/purging type (ANp), and F50.1/307.59 Other Specified Eating or Feeding Disorder type Atypical AN (OSFED-AN) for cases meeting DSM-5 definition of atypical AN [1] (p. 353). For studies published before DSM-5, diagnostic codes F50.8/307.59 were also included if referred to atypically presenting AN classifiable as OSFED-AN according to DSM-5 criteria.

I2: Studies published in English, Spanish, or Catalan.

I3: Studies providing quantitative data on the prevalence of psychiatric diagnoses and/or symptoms in siblings of patients diagnosed with AN according to the I1 criterion.

I4: Full-text availability.

Exclusion criteria:

E1: Studies involving siblings of individuals diagnosed with other EDs or with somatic/mental health conditions involving food, nutrition, or body image (e.g., diabetes, body dysmorphic disorder).

E2: Studies including half siblings of individuals diagnosed with AN according to the I1 criterion if data referred to full and half siblings is not clearly segregated or distinguishable.

E3: Studies reporting exclusively qualitative data on siblings’ experiences.

E4: Case reports, commentaries, book chapters, conference abstracts, and incomplete studies.

E5: Non-human studies.

E6: Studies published in languages other than English, Spanish, or Catalan.

### 2.3. Study Selection and Data Extraction

**Figure 1:**
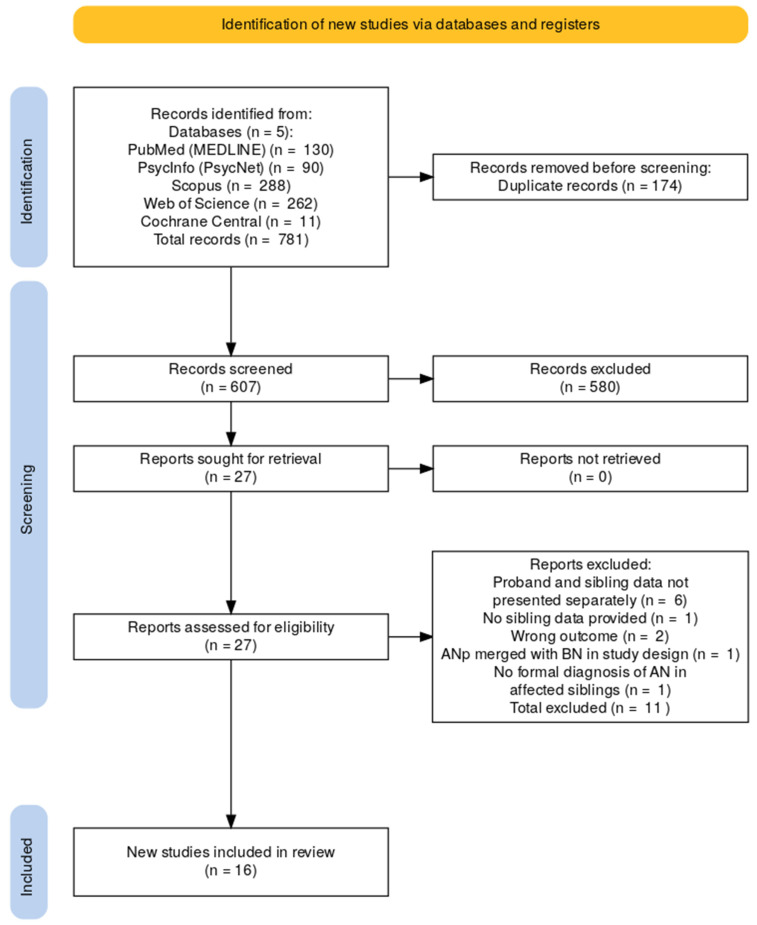
Flow diagram of the systematic review, presented as recommended by the PRISMA Statement Guidelines.

Search results were managed using Rayyan.ai software between 4 June and 28 July 2025 [78]. A total of 781 records were retrieved from five databases. After management of 205 potentially duplicate records detected by the software, 174 real duplicates were confirmed and removed. The remaining 608 articles underwent blinded title and abstract screening by two independent reviewers (E.T.-V. and I.F.). A very high interobserver reliability was observed (97%). Conflicting records were unblinded and reexamined by both reviewers together: consensus was eventually reached in all cases, leading to exclusion of 580 records. Full texts for the remaining 27 articles were retrieved and assessed by a single reviewer (E.T.-V.). Detailed reasons for record exclusion are included in Figure 1.

### 2.4. Assessment of Quality and Risk of Bias

General quality assessments were conducted following the nine-question systematic evaluation tool developed by Hawker et al. [79]. The choice of this tool was guided by the aim of facilitating comparison between our results and those of the most recent topic-related systematic review [32], which presents an excellent synthesis of available data on qualitative and/or phenomenological experiences of siblings of patients with an ED. For each article, a total quality score ranging from nine (very poor) to 36 (good) was computed based on the answers to a series of individual items assessing different quality dimensions. Risk of bias was estimated by means of the Risk of Bias in Non-randomized Studies of Interventions (ROBINS-I) tool, which is freely accessible online on the Risk of Bias Tools website [80,81]. The ROBINS-I was developed in 2016 by the Cochrane Bias Methods Group and the Cochrane Non-Randomised Studies Methods Group. To the best of our knowledge, it is the most exhaustive instrument currently available to assess risk of bias in observational studies; however, according to its authors, it is intended for use particularly in individual non-randomized studies examining the effect of an intervention on an outcome [80]. In accordance with the objectives of our review, we expected to retrieve mostly observational studies, not necessarily including intervention. Due to this limitation, the ROBINS-I could only be partially applied to the articles included in our review, since we were able to apply items referring to outcome measurement and reporting but not those referring to intervention.

Besides the previously mentioned considerations, it shall be underlined that quality and risk of bias assessments were intended as auxiliary procedures contributing to better contextualization of the pieces of evidence retrieved in this review. Thus, given the expectable scarcity of literature relevant to our purposes, prior to performing any search we decided against setting a minimal quality and/or bias risk threshold that would potentially exclude articles failing to prove their proficiency in such terms while still containing information of significant epidemiological and clinical value.

### 2.5. Operationalization and Application of a Sex/Gender Perspective to Study Assessment and Outcome Analysis

As previously argued, one quality dimension particularly relevant to the purpose of this review concerns the use of a sex/gender perspective in study conceptualization, design, data analysis, and interpretation. Numerous guidelines encourage the adoption of such a perspective in research and outline ways of operationalizing it—for example, formulating gender-sensitive hypotheses, promoting sex/gender-diverse research teams, using appropriately representative samples, collecting data on sex and gender, considering sex/gender as a key determinant of health and well-being when analyzing results, providing sex/gender-informed interpretations, and engaging in sex/gender-sensitive dissemination and clinical translation practices. However, to the best of our knowledge, no validated standardized instrument is currently available to systematically assess the quality of a research study from a sex/gender perspective.

Given this gap, a comprehensive expert analysis was performed by a clinician with formal expertise in intersectional sex/gender (E.T.-V.). This analysis was guided by several domains of inquiry, which were systematically applied to each article at two levels:

First level: Descriptive

Based on the analysis provided by the authors:

(a) Is sex/gender considered in study conceptualization, design, data collection, data analysis and/or result interpretation (discussion), and to what extent? If sex/gender is absent in some research process, is this decision commented on and/or justified? Example: Let’s think of a study only including female participants in the sample without explicitly basing this decision on epidemiologic, clinical, logistic, or other reasons.
(b) Are results analyzed and interpreted in a sex/gender-sensitive or any kind of sex/gender-informed manner? That means observing whether sex/gender is analytically considered in the study, only descriptively considered, or not considered at all. Example: Let’s imagine a paper that includes an appropriately sex/gender-diverse sample and reports outcomes separated by sex/gender while not considering sex/gender in study design and discussion of findings, a situation in which the study might appear internally valid yet poorly contextualized and therefore difficult to work with translationally.
(c) Are results discussed in the context of the sex/gender sociopolitical system (and/or other systems that might intersect with it such as ethnicity, socioeconomic status, age, …)? Example: Let’s consider an article that considers sex/gender along research question and study design, data collection and analysis, providing a gender-sensitive discussion of its findings but failing to identify and/or hypothesize a potential intersection between sex/gender and age in the Discussion and Conclusions Section.
(d) Are study limitations identifiable from a sex/gender perspective mentioned in the article, along with their potential effects in terms of decreased quality of research and ethical implications for people who will receive the impact of this research? Example: Let’s picture a paper that only includes female subjects and implicitly roots this decision on gender stereotypes about patients and/or women and girls in general, with a choice of outcomes and instruments that align with such conceptual framework. The article does not consider sex/gender in data analysis, outcome presentation and result discussion, besides stating that the whole sample was female. The authors include a good limitation section in the discussion where they mention risk of bias based on the selection procedure, yet do not critically report their work’s risk of bias from a sex/gender perspective.

Second level: Analytical

Based on the data/information presented in the article:

(a) Could the provided results be potentially analyzed and interpreted in a sex/gender-sensitive or any kind of sex/gender-informed manner? Example: Could sex/gender be analytically considered in the study based on the information available to the reader?
(b) Could the provided results be potentially discussed in the context of the sex/gender sociopolitical system (and/or other systems that might intersect with it such as ethnicity, socioeconomic status, age, …)? Example: Does any potential sex/gender pattern emerge from observation of data that authors fail to identify and/or comment on?
(c) Are there any potentially problematic uses or omissions of sex/gender detectable in study conceptualization, design, data collection, data analysis and/or result interpretation (discussion) to a trained reader eye? Example: Is sex/gender considered conceptually but not empirically in the study, or vice versa? Is the study design biased and/or sex/gender unsensitive? Do authors engage in potentially problematic uses of sex/gender when discussing study results, such as interpreting them in line with gender stereotypes and/or normalizing certain symptoms, attitudes or behaviors when they appear in males while pathologizing them when they appear in females or vice versa?

Global sex/gender perspective assessment results obtained after applying each level of analysis to each article were expressed in the form of a Likert-like scale with the symbols + (high potential for improvement), ++ (considerable potential for improvement), +++ (moderate potential for improvement) and ++++ (low potential for improvement).

## 3. Results

### 3.1. Core Characteristics of Reviewed Studies

Sixteen studies, published between 1983 and 2022 in the European (56.2%), North American (31.3%) and Australian (12.5%) contexts met all inclusion criteria and were therefore included. Table 2 presents the core characteristics of the reviewed studies.

Ten (62.5%) of the papers were classified as high quality (quality assessment score 28 or higher), six (37.5%) as medium quality (quality assessment score between 19 and 27), and none as low quality (quality assessment score 18 or below). Quality assessment scores ranged between 19 and 36. All included studies were classified with a low to moderate risk of bias attending to outcome-related dimensions. Nine (56.3%) of the articles had a control group, which was composed of healthy controls in six cases and community controls in the remaining three cases.

Regarding study design, all studies were descriptive, observational, and cross-sectional for the purposes of this review, including two (12.5%) retrospective studies. Eight of the studies (50%) corresponded to case–control designs.

Descriptively speaking, all the articles included sisters and nine (56.3%) also included brothers of patients with AN. Two papers (12.5%) were all-female twin studies, one was limited to monozygotic pairs and one included monozygotic (75%) and dizygotic (25%) pairs. Of the nine studies including mixed samples of sisters and brothers, four (44%) presented data separated by sex/gender (one of them as Supplementary Material [94]). However, only four papers (25%) contained any sort of analytical insight guided by or relevant from a sex/gender perspective when presenting and/or discussing study outcomes, and none explicitly included a sex/gender perspective in study conceptualization and/or design. In contrast, only five of the articles (31%) mentioned sex/gender perspective problems among their limitations, though strikingly one of them seemed to consider that having included both female and male participants was a limitation [86].

On an analytical level, most of the papers (87.5%) contained potentially problematic uses or omissions of sex/gender identifiable through critical reading by an expert. Of the 12 studies in which authors had provided an analysis and/or discussion of results not guided by a sex/gender perspective, a post hoc analytical application of such perspective that allowed us to nuance, expand, deepen and/or even challenge the original conclusions was possible in three cases (25%, representing 18.8% of all studies). The year of publication did not seem to influence the likelihood and/or extent of consideration of a sex/gender-informed approach, nor did the sex/gender of the authors when disclosed.

Age-wise, three (18.8%) articles studied adolescents, ten (62.5%) studied young adults, and one studied adult pairs with a chronic AN diagnosis in one of the siblings. The remaining studies were conducted in large nation-wide cohorts.

Table 3 presents study quality assessment results overall (Hawker’s tool) and specifically from a sex/gender perspective.

### 3.2. Analysis of Outcomes Reported: Findings Derived from Large Registry-Based Diagnostic Studies Versus Smaller Clinical or Questionnaire-Based Studies

An important conceptual remark must be made regarding methodological heterogeneity before proceeding any further in discussion of the results. Only two articles (12.5%) were based on formal clinician-made diagnoses following international diagnostic categorical classification systems (ICD/DSM), the same two articles that consisted of large registry-based diagnostic studies [91,94]. Another article reported diagnostic data as well, though in the form of self-reported lifetime diagnoses following the SCID, with no direct intervention of any clinician [92].

Most of the studies included in this review reported dimensional symptom elevations, which represent vulnerability to disease but not clinician-diagnosed psychiatric disorders. Though related, the outcomes presented by these studies (absolute or relative elevations in certain symptoms or syndromes) cannot be considered in equivalent terms with formal psychiatric diagnoses.

That being said, among the smaller dimensional and/or psychometrically based studies, five used dimensional standardized psychopathological evaluations of siblings, including those composing the Achenbach System of Empirically Based Assessment (the Child Behavior Checklist—CBCL and the Youth Self-Report—YSR for individuals aged 6 to 18; the Young Adult Behavior Checklist—YABCL and the Young Adult Self-Report—YASR for adults aged 18 to 30), the Symptom Checklist-90—SCL-90, and the Strengths and Difficulties Questionnaire—SDQ. Other articles used validated psychometric instruments and/or somatomorphic measurements to assess specific symptoms (e.g., perceptual body size distortion, disordered eating, depressive symptoms, anxiety symptoms, emotional dysregulation), temperament/personality traits (e.g., perfectionism, harm avoidance, self-directedness), and neuropsychological abilities (e.g., cognitive flexibility, complex facial emotion recognition, attentional bias to social stimuli). Surprisingly, none of the papers used validated questionnaires to assess exposure to traumatic events or posttraumatic stress symptoms: only one study explored the prevalence of adverse life events with an ad hoc questionnaire. Overall, nine (56.3%) papers explored internalizing symptoms, five (31.3%) explored externalizing symptoms, and eight (50%) explored disordered eating and/or body image problems, mainly perceptual distortion and body dissatisfaction. Psychopathology (either categorically or dimensionally operationalized) and other mental health challenges of siblings compared to AN probands and/or controls are the main outcomes of this review and will be described in detail in Section 3.3.

Attachment and parental bonding were also measured in some of the studies, as were other outcomes marginal to the scope of this review such as stigma, perceived social support, and quality of life. Such psychosocial outcomes are complementary to the main ones and will be briefly described in Section 3.4. From a risk factor perspective, two of the studies (12.5%) included a specific psychometric instrument to assess non-shared environment in siblings, the Sibling Inventory of Differential Experiences—SIDE, and one included systematic interviewing on ED risk factors by means of the Oxford Risk Factor Interview for Eating Disorders—ORFI. Additionally, besides psychopathological and other psychometric data, one study included a genetic vulnerability domain with prevalence of relevant polymorphisms in serotonin receptors 5HT2A and 5HT2C, dopamine receptor DRD4, and catechol-O-methyltransferase (COMT) gene in sisters discordant for the diagnosis of AN. However, due to the relatively small sample size, this study failed to identify any significant differences between participants.

### 3.3. Psychopathology and Other Mental Health Challenges in Healthy Siblings Compared to Sick Siblings and/or Controls

Given the heterogeneity and complexity of findings described, in Table 4 we summarize the review’s main findings organized by outcome categories based on evidentiary weight, ranging from the most robust outcomes (clinically diagnosed psychiatric disorders) to the moderately or least robust (elevated scores in dimensional syndromic assessments or above cut-off scores in psychometric measurements of particular symptoms or symptom sets).

#### 3.3.1. Formal Psychiatric Diagnoses

The highest quality studies—those reporting lifetime prevalence of formal psychiatric diagnoses in large nation-wide population cohorts—reported an increased risk of several mental health disorders in siblings, particularly but not limited to those characterized by internalizing symptoms (mood disorders, anxiety disorders) [91,94]. Specifically, siblings of AN probands were significantly more likely to have a lifetime diagnosis of AN, affective disorder, anxiety disorder, obsessive–compulsive disorder, and/or personality disorder than siblings of control probands [91]. In this study, substance use disorders were the only group of disorders considered that did not show an increased prevalence in siblings of AN probands. Nonetheless, in another study exploring substance use dimensionally with the Achenbach System of Empirically Based Assessment, findings indicated that despite AN probands’ and siblings’ prevalence of drug use being very similar, in the case of siblings substance use was much more strongly and consistently linked to psychopathology (internalizing, externalizing and total) [86]. Another large cohort study, including two nation-wide cohorts, concluded that siblings of patients with a lifetime diagnosis of typical or atypical AN were at a significantly increased risk of a lifetime diagnosis of schizophrenia, particularly males who had a sister with AN [94].

#### 3.3.2. Dimensional Psychopathology and Psychometric Assessment

Though methodological heterogeneity was remarkable among the studies, many of them looked at the same outcomes and some even used the same measurement tools, therefore making result comparison slightly easier. For instance, the CBCL/YSR, the YABCL/YASR, the SCL-90, and the SDQ were consistently used in many papers working with a dimensional framework to assess internalizing, externalizing, and total psychopathology in siblings, probands and (if included) controls, though not always with matching results [82,83,84,89].

Overall, studies of this kind tended to conclude that siblings were more similar to controls than to AN probands, except in certain subconstructs of apparently limited clinical relevance [64,66,83,90]. Siblings did not seem to be significantly more perfectionist than controls, neither at present nor in the specific form of obsessive–compulsive traits in childhood [84,88,90,92]. However, on a clinical level, obsessive–compulsive disorder and symptoms were significantly more prevalent in siblings compared to controls [83,91].

Studies comparing internalizing and externalizing psychopathology in AN probands, siblings and controls tended to find increased depressive and anxiety symptoms in siblings compared to controls, although more consistently in the case of anxiety symptoms than in that of depressive symptoms [83,89,90]. There is weak evidence that siblings could present more difficulties in emotion regulation compared to controls, though this has only been reported at trend level by a single study [87]. Evaluated with the SDQ, siblings reported significantly more emotional difficulties, hyperactivity/inattention problems, peer problems and total difficulties than general population, with no differences in conduct problems and prosocial behavior [93]. Notably, the hospitalization of the AN proband was reported to significantly increase emotional, hyperactivity/inattention, and total symptoms experienced by the sibling, according to the same study [93]. When assessed with the Achenbach System of Empirically Based Assessment, siblings scored lower in internalizing and total psychopathology than AN probands but higher than the general population in externalizing psychopathology when parent-reported [93]. Another study, with a retrospective design, found that all symptoms of internalizing and externalizing nature were more reported in the sibling who was later to be diagnosed with AN compared to the other sibling, with larger size effects for internalizing problems. In this study, which included a group of sibling pairs discordant for the diagnosis of BN, siblings of AN patients were found to present lower levels of internalizing, externalizing and total psychopathology compared to siblings of BN patients, though statistical comparison was not provided [82].

Papers exploring disordered eating and/or body image problems in siblings generally reported that siblings scored significantly and consistently much lower than AN probands in such domains, as expectable [76,83,85,88]. However, one article involving sisters currently meeting all criteria for AN, sisters with weight-restored AN, and healthy sisters found that healthy sisters scored more similarly to weight-restored patients than to controls in ED symptoms as measured with the Eating Disorder Examination Questionnaire—EDE-Q [90]. Moreover, in another study including a relatively small sample of sister pairs apparently discordant for the diagnosis of AN, authors found that 9.5% of the non-diagnosed sisters scored within the anorexic range on the Dieting subscale of the Eating Attitudes Test (EAT). These sisters were therefore clinically interviewed by clinicians, who confirmed the existence of a strong drive for thinness. Though the article states that these cases received follow-up, it remains unclear whether they met diagnostic criteria for AN (which is why we did not include this study in Section 3.3.1). The authors suggest that psychometric screening tools such as the EAT might not be accurate enough to detect subtle and/or atypical cases [64]. Another study evaluating eating attitudes and behaviors with the Eating Disorders Inventory-2—EDI-2—found that, while siblings scored significantly lower than AN probands, they also tended to score higher than controls in the domain of body dissatisfaction and—surprisingly—lower than controls in the domain of bulimia [83]. Despite scoring significantly lower than AN probands in disordered eating, in this particular study, sisters scored much higher than brothers [76]. Last but not least, within this group, a single paper examining perceptual body image distortion, self-ideal body discrepancy and negative body evaluation comparing female and male AN patients, siblings and controls found that, while sisters and female controls scored similarly, brothers tended to overestimate their proportion of body fat compared with male controls, who slightly underestimated it (this comparison was significant at trend level) [66]. Despite this interesting finding, neither brothers nor sisters seemed to have more body image problems than controls in this study, despite exposure to the symptoms of AN probands, who showed severe abnormalities in all considered variables. Strikingly, this is the only study in the review including male AN probands.

### 3.4. Impact of AN on Family System and Sibling Subsystem Functioning

The impact of AN on familial functioning and quality of life, particularly on the sibling subsystem relationship, was perceived very negatively by siblings in the reviewed studies, where siblings reported significantly higher parentification rates compared to controls [76,85,89]. One of these papers described that ED symptoms displayed by the AN proband had a greater negative impact on siblings if the whole family system was perceived as more dysfunctional [86]. Moreover, in this study, siblings perceived that the greater the level of familial dysfunction was, the lesser social support they received, particularly in the form of negative societal attitudes directed not only towards the individual suffering from AN but also to the whole family system [85]. In addition, in siblings, more negative perceptions about the AN proband appeared to be associated with higher levels of depressive and anxiety symptoms in one study [89]. Regarding differential sibling experiences (non-shared environment), premorbid parental treatment and peer characteristics were reported to be perceived as similar by both siblings and AN probands, even though the latter’s perceptions of premorbid dysfunction of the sibling subsystem were generally more negative in terms of them (future AN patients) feeling more jealous of their siblings and overestimating their attractiveness and popularity. Concordantly, siblings reported feeling significantly less jealous of AN probands than vice versa in these studies [85,88].

## 4. Discussion

This article aimed to systematically review current quantitative and specific evidence on the prevalence of mental health psychiatric symptoms, diagnoses and other mental health challenges in male and female siblings of patients with AN and analyze it with a particular interest on integrating both internalizing and externalizing problems and critically assessing existing evidence with the guidance of a sex/gender perspective.

### 4.1. Amount and Overall Quality of Existing Research

Despite the background literature suggesting that a very limited amount of research meeting our inclusion/exclusion criteria was expectable, we were able to locate sixteen papers fit for inclusion, most of them rated as high-quality and none below medium quality by a widely used overall quality assessment tool. These quality assessment results are consistent with those obtained by Heneghan et al. in their review [32]. Articles in our review obtain more modest quality scores overall, though with higher variability, when compared to the other review. A possible reason for this is that our review is aimed toward studies reporting quantitative data on diagnoses, syndromes and symptoms, which appear to be less common and more methodologically demanding than qualitative studies: from this perspective, higher scores might be harder to achieve for these studies. However, it is reasonable to conclude that the overall quality of the papers included in this review is satisfactory. Regarding the risk of bias in outcome measurement and reporting, as measured with the ROBINS-I tool, all studies included in our review were estimated to be at a low to moderate risk, which is also an acceptable result. In this paper, sex/gender perspective is conceptually framed as a guiding analytical lens for a deeper, critical and more contextually engaged understanding of clinical and translational research, and parallelly operationalized as a strategic tool for its comprehensive quality assessment. Insights and considerations related to sex/gender perspective will be discussed later in a specific subsection, since they connect with one of the review’s specific aims previously listed.

Despite overall adequate formal assessment results, the first main finding of this review is that only a few studies exist providing systematically collected, quantitative and specific data on psychiatric diagnoses and symptoms in siblings of patients with AN, obtained with standardized clinical interviews and/or psychopathology assessment tools, either categorial or dimensional. Moreover, studies reporting formal psychiatric diagnoses are even scarcer. This confirms the existence of a gap in current knowledge on siblings’ mental health starting off at the most basic level, that is, epidemiologic data such as prevalence and incidence, particularly of formal clinician-made psychiatric diagnoses.

### 4.2. Discussion and Framing of Study Results

Overall, the very few studies published up until the present day based on clinician-made formal psychiatric diagnoses conclude that siblings of AN probands are more likely than controls and the general population to be diagnosed with an affective disorder, an anxiety disorder, obsessive–compulsive disorder, schizophrenia, a personality disorder, and/or an eating and feeding disorder, including AN [91,94].

Compared to AN probands, studies based on subclinical vulnerability suggest that siblings tend to experience less total, internalizing, and externalizing psychopathology, despite both AN probands and siblings scoring apparently lower than controls in externalizing problems [82,86]. Compared to controls, however, siblings might be at an increased risk of displaying elevated internalizing (depression, anxiety, emotional difficulties, inattention) and externalizing (hyperactivity, peer problems) symptoms when assessed with dimensional tools [90,93]. These results seem compatible with previous literature reporting increased risks for EDs, mood and anxiety disorders, and other emotional difficulties in siblings [3,14,28,31].

Interestingly, siblings tend to score closer to controls than to AN probands when psychometrically assessed for problematic eating attitudes and behaviors and body distortion/dissatisfaction [64,66,76,83,85,88,90], but obtain higher prevalence of ED diagnoses when formally assessed by clinicians [64,91], a finding also consistent with the previous literature [54]. This points to a potential limitation of questionnaire-based screening strategies in this population, as mentioned in one of the reviewed papers [64], but should also be interpreted in the context of relatedness but non-equivalence between formal clinician-made diagnoses and results of patient- and/or parent-reported dimensional, psychometric tools.

Obsessive–compulsive disorder, symptoms and traits deserve further comment since, in our review, such problems seem to emerge as particularly relevant for the siblings’ pathways to mental disease/distress. On the one hand, siblings could be at an increased risk of obsessive–compulsive symptoms and disorder diagnosis [83,91]. On the other hand, the noneating-related obsessive–compulsive symptoms displayed by the proband either in the context of AN or in that of comorbidity seem to greatly impact the healthy sibling’s perceptions of family dysfunction and impaired quality of life in one study [76]. Remarkably, in one of the mentioned studies, authors suggest that the increased presence of obsessive–compulsive traits in the siblings might be mitigated by their apparently healthier patterns of personality and personal functioning [83], an idea that seems consistent with the findings of other studies included in this review [83,85,87,88,90,92] and also with background literature referring to chronic and/or life-threatening childhood disease [33,34]. Conversely, in another study, the presence and degree of functional impairment of obsessive–compulsive traits during childhood and adolescence, as retrospectively self- and parent-reported, do not appear to be increased in siblings compared to controls [84]. According to this study, obsessive–compulsive traits in childhood might be significantly more prevalent and severe in siblings who are later to be diagnosed with AN compared to both siblings who are to remain healthy and controls. In addition, this study describes that the presence of childhood obsessive–compulsive traits could be associated with a significant increase in the risk of receiving a future AN diagnosis and, once the diagnosis is established, the number of traits could positively correlate with greater severity of disordered eating, body image distortion and depressive symptoms [84].

Moreover, and in addition to a potentially increased vulnerability to psychopathology in the terms and within the conceptual considerations previously discussed, siblings generally perceive AN to have a negative and severe impact on their personal and familial quality of life, particularly in cases exhibiting greater familial dysfunction and/or pathology. In such cases, siblings report perceiving less social support from the community and more stigma, both directed not only toward the AN proband but also to the whole family system [76,85,89]. This is consistent with the previous literature [31,32]. While premorbid parental and peer-to-peer relationships are similarly perceived by both AN probands and siblings, siblings seem to report less premorbid dysfunction of the sibling subsystem, particularly in terms of jealousy [88]. These findings point to the importance of non-shared environmental factors, particularly differential sibling experiences, in the pathways to health/disease regarding AN. A compatible explanation could be that the sibling perceiving themselves less favorably is the most likely to become the AN proband, even though objective differences between siblings might be few, as siblings report qualitatively similar premorbid experiences with parents, peers, goals and achievements. Consistently, it has been reported that greater sibling conflict could be a predictor of future disease onset [43,44]. On a postmorbid level, however, the sibling’s perceptions about the AN proband seem to generally worsen, and more negative perceptions on the AN proband could arguably increase siblings’ risk of anxiety and depressive symptoms [89]. This is also in line with previous findings on negative affect, emotional burden, and difficulties faced by siblings [32,45,73,75].

It should be noted that we are aware of the existence of other quantitative and/or mixed-methods sibling studies focusing on cognitive, emotional, or stress-related domains, some of them recently published [95], that were not included in this review as they were considered to fall outside of its scope during study selection procedures, in careful application of the predefined inclusion/exclusion criteria as interpreted by the reviewers responsible for such procedures. We remind readers that exact inclusion and exclusion criteria are detailed in Section 2.2 of the Materials and Methods Section.

### 4.3. Insights and Considerations Related to Sex/Gender Perspective

From a sex/gender perspective, understood as both a conceptual framework and an analytical tool with a high potential to increase ethical and epistemological quality of research [48,49], the results of our review are quite discouraging. Slightly over half of all presented studies included brothers of patients with AN, while all included sisters. Twin studies were limited to sisters, even in the case of dizygotic twins. In our view, this selection bias is not justifiable by the higher prevalence of AN in females nor the relatively high heritability of the disorder.

None of the studies in this review specifically included a sex/gender perspective in their conceptualization and design. On a result level, a minority of one in four papers included sex/gender in the outcome analysis and/or discussion, and most of them presented potentially problematic uses or omissions of sex/gender that were neither acknowledged nor addressed by the authors, hence favoring the emergence of sex/gender biases.

The main potentially problematic points identified in this review included (a) exclusion and underrepresentation of male siblings, (b) limiting sex/gender analyses to a descriptive level while failing to contextualize findings within the intersectional sex/gender sociopolitical framework, and (c) considering sex/gender when addressing certain symptom domains, such as body image concerns and disordered eating patterns, while ignoring or overlooking this factor when examining other mental health problems, particularly externalizing psychopathology.

In a second-level attempt to critically appraise existing literature from a sex/gender perspective, we attempted to examine the data and analyses provided by the authors to identify potential opportunities for expanding, nuancing, deepening and/or challenging their original conclusions. To the best of our efforts, this was only partially feasible in most of the articles. The most common limiting factors at this point were the authors’ decisions to (a) exclude male siblings, (b) not provide sibling outcome data disaggregated by sex/gender, and/or (c) provide none or insufficient insights on sex/gender context for the reported data.

Therefore, besides the already-mentioned scarcity of available evidence and particularly of evidence based on formal clinician-made diagnoses, another key gap identified by this review is the design and production of potentially problematic research from a sex/gender perspective. This is a conceptual, transversal limitation with important epistemic, ethical, and clinical implications. In this sense, it should be underlined that we are pointing to a structural problem that extends beyond the mere exclusion of male siblings and the consequent reduction in the studies’ potential to identify possible sex/gender patterns.

Before we proceed any further in this subsection, we must stress that the main contribution of this review in terms of the use of a sex/gender perspective lies in documenting systematic absences and biases in the literature, rather than in identifying latent sex/gender patterns. However, the systematic application of a two-layered sex/gender analysis to the reviewed articles allowed us, in particular cases, to identify potential productive tensions in the original articles’ result analyses and discussions that could be further explored in future research conceived and analyzed entirely from an intersectional sex/gender perspective. In the next paragraphs, we will briefly discuss a couple of anecdotic examples in hopes that they can be useful to illustrate this point, particularly regarding the emphasis on intersectionality.

Related and complementary to the systematic deficiencies in the use of a sex/gender perspective pointed by our review, our critical sex/gender analyses suggest that the existing literature could be more sex/gender-sensitive when assessing adult relatives (parents) of AN probands than their closer-in-age siblings. A possible reason for this could be that adult caregiver roles are more explicitly gendered, particularly in conventional heteropatriarchal families, and therefore researchers sharing cultural contexts with patients and families may implicitly normalize and expect gendered parental responses. In this sense, if interpreted with a sex/gender perspective, findings reported in a couple of the reviewed studies could be compatible with the hypothesis of fathers showing a lower level of personal implication and competence regarding their children’s emotional difficulties, particularly of non-AN siblings, an observation supported by our clinical experience [86,93]. In addition, another paper included in this review reports a differential pattern of mental distress expression in mothers and fathers of patients with AN, with mothers being more prone to internalizing symptoms, disordered eating and structural psychopathology (personality disorders) and fathers being more likely to engage in problematic substance use [91]. Such differences could be explained by sexual dimorphism but also by—and, in any case, in interplay with—societal gender norms regarding acceptability of mental distress expressions and gender bias in clinician assessments of mentally distressed individuals.

Besides illustrating the potential effect of applying a sex/gender perspective to outcome analysis and discussion, these examples suggest how sex/gender differences and stereotypes might intersect with other dimensions, such as developmental processes and ageism. However, interesting as these potential interactions between sex/gender and age might be in terms of unraveling siblings’ pathways to psychopathology and mental suffering, we shall explicitly caution against assuming developmental continuity across childhood, adolescence, and adulthood in such processes.

Perhaps due to the problems with sex/gender perspective, in our review we could not find any sound evidence supporting the reversed sex/gender pattern suggested by some previous works [29].

### 4.4. Limitations

Despite careful adherence to PRISMA quality standards [77], this systematic review is not free from certain limitations that must be considered for a proper understanding and use of its results. Some of these limitations relate to the methodology used in the review itself, while others stem from limitations of the original articles included in the review.

#### 4.4.1. Limitations of the Review

First off, the search covered five databases, which we consider reasonable, though not exhaustive; therefore, some relevant papers may have been missed. Nonetheless, we used the largest and most widely consulted databases in the field, and all studies identified during full-text screening could be successfully retrieved, despite not all of them being open access.

Secondly, general risk of bias assessment was only partially applicable because the high-quality tool we selected is designed for observational studies involving an intervention, whereas most of the included studies did not meet this criterion. The rationale for selecting this tool is detailed in the corresponding section. We addressed this limitation by further assessing quality and risk of bias from a sex/gender perspective.

Thirdly, while title and abstract screening were conducted independently by two blinded reviewers, full-text screening, data extraction and sex/gender analyses were performed by a single reviewer and subsequently reviewed in detail by the research team.

#### 4.4.2. Limitations of the Included Studies

Several sources of bias are detectable in the reviewed studies. The most relevant ones from our perspective are discussed in detail in this section, along with their potential effects in terms of inflation or distortion of observed associations.

*Geopolitical bias*: All included articles were carried out in the European, North American and Australian regions. Even among the excluded records, there was a clear over-representation of these regions. We currently remain unaware of the mental health status and processes of the siblings of patients with AN from most of the world’s territories and communities.

*Selection bias*: In many studies, sampling was incidental, resulting in a decrease in external validity and applicability of obtained conclusions. In addition, some studies limited participation to only one sibling, typically the first to respond or the closest in age to the proband. This might contribute to underestimating sibling psychopathology, since the healthiest siblings might be more able, prone, and fast to participate. Importantly, this may also hamper detection of potential age-related patterns and of subclinical or prodromal symptoms in youngest siblings. A particularly relevant form of selection bias is exclusion of male siblings: as already discussed, almost half of the studies included only sisters. This leads to the existence of a knowledge gap in brothers’ specific mental health problems and challenges, even though brothers represent half of the sibling population. Additionally, excluding brothers limits the potential for detecting possible sex/gender-related patterns and might result in overestimation of certain symptoms (the ones more prevalent in females) in general knowledge regarding siblings’ mental health status. Regarding participant profile, even though participant age, time of exposure to treated/untreated disease, and degree of severity and chronicity varied considerably among studies, we observed a tendency towards recruitment of relatively stable outpatients, likely because of methodological convenience. Since AN typically begins during late childhood or adolescence, older patients are also more likely to be recruited, as are chronic patients exposed to longer times of treated disease and/or contact with health services. Accordingly, most of the reviewed studies included probands at their late adolescence or early adulthood, and none included early-onset AN probands (aged 14 or below). Relatively high participant age could lead to underreporting mental health challenges typically experienced by younger siblings and/or symptoms typically presenting at younger ages. Conversely, overrepresentation of probands tending to chronicity and/or in a relatively stable condition after receiving several months of treatment might allow time for siblings’ psychopathology to emerge and progress to detectable levels, while at the same time encumbering the detection of subclinical or prodromic phases, which are stages of particular interest in child and adolescent psychopathology.

*Recall bias*: Retrospective designs and reliance on parent reports might contribute to underreporting psychopathology in siblings and controls compared to AN probands. Parental attribution bias could also play a role in distorting results in this sense. Similar behaviors could have been interpreted differently by parents when displayed by AN probands or by healthy siblings. Such differences could be quantitative (e.g., perceiving difficulties as more intense or severe in AN probands) but also qualitative (e.g., interpreting the same phenomenon as a symptom when observed in the AN proband and as developmentally appropriate when observed in the healthy sibling). To complicate matters even further, parental attribution bias could arguably be less prevalent in more educated families, in parents of certain professional groups, and in those more exposed to appropriate professional counseling and/or psychotherapy.

*Outcome heterogeneity*: Many studies limited considered outcomes to AN core symptoms and/or associated features (e.g., eating attitudes and behaviors, body image concerns, negative affect, perfectionism). This is likely to result in the underestimation of other relevant mental health problems, particularly externalizing symptoms. Unexpectedly, however, posttraumatic symptoms and/or exposure to potentially traumatic events were generally missing from the studies’ outcomes. Moreover, as already underlined previously in this paper, some of the studies included in this review were based on clinically diagnosed psychiatric disorders, while others were based on subclinical vulnerability, e.g., dimensional symptom elevations. Though related, these outcomes are qualitatively different and should not be considered equivalents.

*Other methodological issues*: In some studies, ad hoc classifications of the probands’ diagnoses (e.g., in restrictive and bulimic syndromes), or obsolete diagnostic classification systems (e.g., DSM-IV or DSM-IV-TR), were potentially challenging in terms of correctly identifying the inclusion of participants of interest comparable to our inclusion criteria. Another relevant limitation was the treatment of sibling data as a secondary or partial outcome in studies mainly focused on other relatives or with different objectives. This was the case for some studies including siblings of patients diagnosed with any ED which failed to provide disaggregated data of siblings of patients with a particular diagnosis of AN for some particular outcomes. Regarding shared-method variance, cross-sectional studies based on psychometric evaluation of several (and sometimes similar) variables by a single rater (often parents or caregivers) could be at particular risk of reaching biased conclusions. Remarkably, few studies had longitudinal designs, and none had a prospective design. These designs are the most appropriate for capturing processes rather than states, processes such as pathways to mental distress/disease, which are of particular interest in stages such as childhood and adolescence, characterized by ontogenetic change.

### 4.5. Strengths and Potentials

Of this review:

- To the best of our knowledge, this is the first systematic review examining quantitative information regarding the prevalence of psychiatric diagnoses, symptoms and/or other mental health challenges in siblings of patients with AN characterized as a significantly at-risk group. We are also unaware of the existence of any other review centered in this outcome for siblings of patients more generally diagnosed with an ED.
- A key strength of our review is the integration of a sex/gender perspective grounded in corpomaterialist and intersectional feminist theory. This framework highlights how the sex/gender of probands and siblings may interact differently with other factors (e.g., family functioning, developmental stage, coping styles, or family body-related practices). Most previous research has not used this lens, leading to limited or biased knowledge focused mainly on sisters’ internalizing and eating-related experiences. Age-related biases may also have contributed to conflating sisters’ experiences with those of mothers, despite distinct developmental pathways in younger siblings.

Of the included studies:

- Most of the included studies scored considerably well in terms of quality and risk of bias. The review included two top-quality studies conducted on large nation-wide cohorts.
- Despite outcome and methodological variability, many of the articles used the same or comparable instruments to measure outcomes of interest, making integration and comparison easier.
- A few studies engaged in sex/gender-informed analyses to some extent and/or allowed post hoc application of a sex/gender perspective in result interpretation.

### 4.6. Conclusions

After completing our systematic review and analyzing its results against the backdrop of the theoretical framework exposed in the Introduction Section, we identify the following knowledge gaps:

- **The current available evidence on psychopathology and other mental health challenges in siblings is limited.** There is insufficient knowledge on the prevalence of psychiatric diagnoses and/or symptoms in siblings of individuals diagnosed with AN, particularly due to the limited number of studies systematically exploring psychopathology in this group with validated clinical interviews and/or comprehensive diagnostic tools that allow reaching a clinician-made diagnosis when needed and identifying prodromic and subclinical stages.
- **Exclusion of male siblings and disregard of externalizing symptoms are the main sources of bias.** Despite scoring more than decently in overall quality and risk of bias, the few existing studies suffer from certain limitations that might arguably place them at risk for bias: (a) there is an underrepresentation of males in the sibling samples, and (b) many studies fail to systematically explore externalizing symptoms and problems not directly related to eating/body image.
- **The existing research is potentially problematic from a sex/gender perspective**. When analyzed with a sex/gender perspective, the quality of the existing research on the matter decreases substantially, therefore revealing a worrisome risk of bias with significant epistemic and ethical consequences that may not be accurately captured by general formal assessment tools. Most of the existing studies fail to meet current recommendations regarding the need to conceptualize, carry out, analyze and report research with a sex/gender perspective, in accordance with overwhelming evidence of sex/gender as a major determinant of health. Production and dissemination of comprehensive, integrated and adequately contextualized knowledge on the mental health processes of siblings of patients with AN should be actively encouraged.
- **While siblings seem to be more similar to controls than to AN probands in terms of subclinical vulnerability, large cohort studies based on formal clinician-made psychiatric diagnoses support the idea of siblings being at an increased risk for psychopathology.** Studies based on self- or parent-reported dimensional psychopathological traits might not be sensitive enough to detect mental health problems in siblings. However, many vulnerability studies interestingly suggest that non-shared environment could play a key protective role in siblings exposed to high burdens of proband disease and familial distress. According to these studies, even in symptomatic domains where they score higher than controls, siblings seem to remain less functionally impaired, at least during initial stages of the disease.

### 4.7. Suggestions for Future Research Directions

The purpose of this review was to provide a theoretical and empirical background to guide future translational research aimed to broaden and further define knowledge on the specific pathways to mental distress and psychopathology in siblings of patients suffering from AN, the most prevalent and life-threatening of EDs, from a sex/gender perspective. Given the current knowledge on (a) the effects of potentially traumatic experiences during the neurodevelopmental period, (b) the centrality of the family subsystem in such ages and particularly of the sibling subsystem, (c) the role of sex/gender as a main determinant of health and well-being experiences, (d) the increased risk of first-degree relatives of patients with EDs due to shared genetics and environment, and (e) the benefits of early detection and treatment in child- and adolescent-onset mental disorders, advances in translational research on siblings of patients with EDs is an absolute clinical and ethical priority.

That being so, we strongly recommend future research endeavors to be strategically pointed towards systematic screening of psychopathology in siblings of patients suffering from AN and other EDs with a specific focus on sex/gender perspective, both from a cross-sectional (prevalence, correlations) and from a prospective (pathways to mental disease/health, challenges and opportunities for mental well-being) standpoint. Advances in this issue will be key to design future proactive, personalized and efficient interventions for mental health promotion and disorder prevention in this specific at-risk group.

In this regard, as previously mentioned in this paper, a recent study underpins that approaches widely explored and proven successful in incipient psychosis, based on actively identifying prodromal symptoms and thus shortening the duration of untreated illness, could also be key to early detection and prognosis improvement in EDs [19]. Particularly, the authors of this study support the concept and application of the chronopathogram, an innovative representation of pathological events as they unfold over time [19]. In light of our results and in concordance with the previous literature on pathways to AN [12,13], this approach seems to us a particularly appropriate and promising tool for prodromal phase detection allowing early strategic intervention on siblings’ and other at-risk individuals’ pathways towards complex, multifactorial and multi-layered mental disorders. In our view, the chronopathogram and other similar methods based on the concept of ontopathogenesis should be further explored in their applied potential as resources for disease prevention and mental health promotion in children and adolescents with increased risk of serious mental disorders. Meaningfully, in our opinion, the potential of this tool in siblings of patients with AN extends beyond early detection of disordered eating and body dissatisfaction but rather facilitates integration of other internalizing and externalizing expressions of mental distress and/or mental health challenges that, if left undetected and untreated, may contribute to an increased risk of a range of psychiatric disorders.

## Figures and Tables

**Table 1:** Factors contributing to increased risk of mental distress and disease in siblings of individuals with AN.

Shared genetics	Heritability of AN and other frequently comorbid conditions (e.g., obsessive-compulsive disorder, affective disorders) Heritability of neurocognitive phenotypes and temperament/personality traits Heritability of bodily features associated with body image and eating patterns (e.g., precocious puberty, obesity) Biological sex (in concordant cases)
Shared environment	Family premorbid attitudes toward food, weight, and appearance Family functioning patterns, parenting style, family values Family lifestyle factors (e.g., diet, physical activity patterns) Shared external influences (e.g., peers, teachers, leisure activities shared with siblings) Shared traumatic experiences (e.g., gender-based violence at home) Obstetric history (in some cases) Socioeconomic status Gender (in concordant cases) Ethnicity Societal beauty standards Diet culture Healthcare system
Non-shared environment	Birth order Parental bonding Non-shared external influences (e.g., peers, teachers, leisure activities exclusive of one sibling) Individual traumatic experiences (e.g., sexual abuse, bullying) Individual lifestyle factors (e.g., drug use, engagement in elite sports) Obstetric history (in most cases) Gender (in discordant cases) Family dynamics after illness onset (e.g., parentification, perceived neglect and isolation) Exposure to sibling’s AN symptoms Access to accurate information about the sibling’s condition and to adequate emotional support

**Table 2:** Core characteristics of reviewed studies.

Study	Design	Siblings	AN Probands	Controls
Adambegan et al. (2012) [82], Austria	Cross-sectional (case-control design), retrospective	n = 37; 100% female; mean age = 25.2 (SD 7.8); mean BMI = 21.6 (SD not provided); with no lifetime history of EDs	n = 37; at least 3 years fulfilling criteria for ANr; 100% female; mean age = 25 (SD 6.6); mean BMI = 19.2 (SD not provided); mean duration of illness 7 years (SD not reported); 47% firstborns	No control group
Amianto et al. (2011) [83], Italy	Cross-sectional (case-control design)	n = 31; 71% female; mean age = 26.3 (SD 7.4); mean BMI = 21.1 (SD 2.6); with no lifetime history of any psychiatric disorder	n = 38; 82% female; mean age = 26.6 (SD 8.3); mean BMI = 16.4 (SD 2)	Healthy controls: n = 50; 70% female; mean age = 24 (SD 2.1); mean BMI = 21.9 (SD 2.7)
Areemit et al. (2010) [76], Canada	Cross-sectional	n = 14; 70% same-gender (female); mean age = of all siblings included in the study (of patients with AN and patients with other EDs) 13.7 (SD 2.1)	n = 13; 100% female; mean age = 14.5 (SD 1.9)	No control group
Benninghoven et al. (2008) [66], Germany	Cross-sectional	n = 38; 66% female; mean age = 21.1 (SD 5.6) for females and 22.2 (SD 6.7) for males; mean BMI = 23 (SD 4.5) for females and 24.5 (SD 5.6) for males	n = 18 inpatients with current active AN; 100% female; mean age = 21.9 (SD 4.4); mean BMI = 15.1 (SD 1.5)	Healthy controls: n = 60; 67% female; mean age = 23.7 (SD 4) for females and 25.2 (SD 4.5) for males; mean BMI = 21.4 (SD 2.5) for females and 22.8 (SD 2.6) for males
Degortes et al. (2014) [84], Italy	Cross-sectional, retrospective	n = 32; 100% female; mean age = 27.8 (SD 8.4); mean BMI = 21.8 (SD 2.4); with no lifetime history of EDs *Assessment remark*: In this group, n = 19 participants were directly interviewed and for the rest (n = 13) maternal reports about childhood traits were obtained	n = 116; 100% female; mean age = 23.2 (SD 6.2); mean BMI = 17.7 (SD 3.7); mean lowest lifetime BMI 15.4 (SD 1.7); n = 61 (52.5%) meeting all AN diagnostic criteria at the time of study	Healthy controls: n = 119; 100% female; mean age = 27.1 (SD 3.9); mean BMI = 21.2 (SD 2.6); all with no personal or family history of ED, axis I comorbidity, alcohol or substance abuse, or use of psychoactive medication
Dimitropoulos et al. (2013) [85], Canada	Cross-sectional (case-control design)	n = 26; 61.5% female; mean age = 24.9 (SD 7.7) *Selection remark:* If more than one sibling was available, the closest in age to the AN patient was the one included	n = 26; 96% female; 58% ANr; mean duration of illness 8.2 years (SD 5.9); mean age = 26.8 (SD 7); mean BMI = 15.1 (SD 1.9)	No control group
Halvorsen et al. (2005) [86], Norway	Prospective (cohort study), but cross-sectional for non-AN siblings’ data, since this group was only assessed at follow-up	n = 31; 55% female; mean age = 24.4 (SD 5.5) *Assessment remark:* These siblings were self-assessed, and 68% were also assessed by at least one of their caregivers (mother and/or father) in comparison with their AN sibling, only at follow-up *Selection remark:* The sister closest in age, or the brother closest in age if the patient did not have a sister, was the one chosen to participate	n = 55 former patients who had met DSM-IV criteria for AN and received psychiatric treatment between 1986 and 1998; 100% female; mean age = at treatment start 14.9 (SD 1.8); mean BMI = at treatment start 15.1 (SD 1.6); mean duration of illness at treatment start 10.9 months (SD 6.5); mean age = at follow-up 23.1 (SD 3.4) *Assessment remark:* These siblings were self-assessed, and 70% were also assessed by at least one of their caregivers (mother and/or father) in comparison with their non-AN siblings	Population data (normalized T-scores were used when comparing the former patients with their siblings)
Kanakam et al. (2013) [87], United Kingdom	Cross-sectional	n = 12 twins regardless of whether they also had an ED diagnosis; 100% female	n = 24 twins with a chronic AN diagnosis including AN-r, AN-p, and atypical AN according to DSM-IV-TR criteria (18 monozygotic and 6 dizygotic); 100% female	Healthy controls: n = 42 from 21 twin pairs (17 monozygotic and 4 dizygotic) 100% female; mean age = 42.6 (SD 12.8); mean BMI = 22.5 (SD 2.6)
Karwautz et al. (2001) [88], United Kingdom	Cross-sectional (case-control design)	n = 45; 100% female; mean age = 27.4 (SD 9.7); mean BMI = 22.4 (SD 3.8)	n = 45; 100% female; mean age = 27.7 (SD 8.5); mean BMI = 17.7 (SD 3.7)	No control group
Maloney & Shepard-Spiro (1983) [64], United States of America	Cross-sectional (case-control design)	n = 21; 100% female; mean age = 24 (SD not provided); mean BMI = 21.5 (SD not provided) *Selection remark:* If more than one sister was available, the closest in age to the AN sister was the one included	n = 21; at least 3 years meeting diagnostic criteria for AN; weight not below 10% normal weight for height/age to avoid starvation effects; 100% female; mean age = 20 (SD not provided); mean BMI = 18.8 (SD not provided)	Healthy controls from another study: n = 81; 100% female; mean age = 21.5 (SD not provided); mean BMI = 22.2 (SD not provided)
Matthews et al. (2021) [89], United States of America	Cross-sectional (case-control design)	n = 34; 70.6% female; mean age = 15.1 (SD 2.2); 23.5% currently receiving psychotherapy (reason unknown); 61.8% younger than AN sibling. *Selection remark:* Only the first sibling recruited from a given family was retained for analysis	n = 34 with current active typical or atypical AN; 93.5% female; mean age = 16.1 (SD 1.7); mean duration of illness 1 year (SD 1)	Community controls: n = 47; 57.4% female; mean age = 14.2 (SD 1.8); 8.5% currently receiving psychotherapy (reason unknown). *Selection remark:* Only the first control subject recruited from a given family was retained for analysis
Phillipou et al. (2022) [90], Australia	Cross-sectional	n = 20; 100% female; mean age = 22.8 (SD 2.9); mean BMI = 23.3 (SD 4)	Group of current AN (c-AN): n = 20; 100% female; mean age = 22.5 (SD 3.1); mean BMI = 16.7 (SD 1.48) Group of weight-restored AN (wr-AN): n = 20; 100% female; mean age = 22.5 (SD 2.8); mean BMI = 21.7 (SD 1.96)	Healthy controls: n = 20; 100% female; mean age = 24 (SD 4.4); mean BMI = 23.4 (SD 3.2)
Steinhausen et al. (2015) [91], Denmark	Cross-sectional (case-control design)	n = 2854 siblings of individuals diagnosed with AN (case probands) identified through the Danish Central Civil Registration Register; 51.4% female	n = 2370 case probands with a lifetime history of AN identified through the Danish Psychiatric Central Research Registry; 92% female	Community controls: n = 7035 control probands matched to case probands on age, sex/gender and region of residence after identification in the Danish Central Civil Registration Register; 92% female + n = 9292 siblings of control probands; 49.1% female
Thornton et al. (2017) [92], United States of America	Cross-sectional (case-control design)	n = 22; monozygotic; 100% female; mean BMI = 22 (SD 2.5); with no lifetime history of AN, including subclinical presentations	n = 22; 100% female; mean age = 31.7 (SD 6.3); mean BMI = 20.9 (SD 1.7)	No control group
van Langenberg et al. (2016) [93], Australia	Cross-sectional assessment of 2 possibly partially overlapped samples at point 1 (diagnosis) and point 2 (after Family-Based Treatment, FBT) **	Study sample A: n = 55; 56.4% female; mean age = 16.4 (SD 4.3) *Assessment remark:* These siblings were both self-assessed and parent-assessed by n = 47 mothers and n = 38 fathers Study sample B: n = 46; 60.9% female; mean age = 16.4 (SD 3.5) *Assessment remark:* These siblings were self-assessed and parent-assessed by n = 64 mothers and n = 24 fathers	Study sample A: n = 46; 91.5% female; mean age = 15.4 (SD 1.7); meeting criteria for AN or OSFED-AN Study sample B: n = 38; 92.1% female; mean age = 16.1 (SD 1.8); meeting criteria for AN or OSFED-AN	Population data from parental and self-reports of Australian boys and girls aged 11 to 17 (n not reported)
Zhang et al. (2021) [94], Sweden	Cross-sectional	All siblings of individuals diagnosed with AN (including atypical AN) identified through the Swedish Multi-Generation Register and the Danish Central Civil Registration Register ***	n = 51,168 individuals diagnosed with AN (including atypical AN) identified through the Swedish Multi-Generation Register and the Danish Central Civil Registration Register; 93% female	Community controls (rest of the cohort); n = 2,004,087

* BMI: Body Mass Index; SD: Standard Deviation. BMI is expressed in kg/m^2^. Ages are expressed in years. ** The proportion of the sample that was assessed twice remains unclear, but the design cannot be considered a pre-post study based on the information provided in the article. Study sample A was assessed at diagnosis and before starting FBT. Study sample B was assessed after completing 6 months of FBT. *** The study also included individuals diagnosed with other EDs and their families, and demographic data is presented all-together, so the exact number of siblings of individuals with AN remains unknown. Information on half siblings was also included in the study, though was excluded from this review in accordance with inclusion/exclusion criteria.

**Table 3:** Study quality assessment results overall and from a sex/gender perspective.

Study	Overall Quality Assessment Score	Quality Assessment from a Sex/Gender Perspective	
		First Level: Descriptive	Second Level: Analytical
Adambegan et al. (2012) [82], Austria	26	+	+
Amianto et al. (2011) [83], Italy	28	++	+
Areemit et al. (2010) [76], Canada	32	++	+++
Benninghoven et al. (2008) [66], Germany	29	+++	+++
Degortes et al. (2014) [84], Italy	27	+	+
Dimitropoulos et al. (2013) [85], Canada	26	++	+
Halvorsen et al. (2005) [86], Norway	31	++	++
Kanakam et al. (2013) [87], United Kingdom	29	+	+
Karwautz et al. (2001) [88], United Kingdom	31	+	+
Maloney & Shepard-Spiro (1983) [64], United States of America	21	+	+
Matthews et al. (2021) [89], United States of America	32	+++	++
Phillipou et al. (2022) [90], Australia	26	+	+
Steinhausen et al. (2015) [91], Denmark	36	+++	++
Thornton et al. (2017) [92], United States of America	19	+	+
van Langenberg et al. (2016) [93], Australia	29	++	++
Zhang et al. (2021) [94], Sweden	36	+++ *	++

* Sex-specific sensitivity analyses of the subsample of full siblings are provided as supplementary material and referred to in the full text.

**Table 4:** Outcome-based analysis and summary of main findings.

Outcome of Interest	Studies Measuring Outcome	Measurement Tools	Plain Summary of Significant Findings
Psychiatric diagnoses	Steinhausen et al. (2015) [91], Denmark	Diagnoses based on the International Classification of Diseases according to the World Health Organization (ICD) registered in nationwide databases	Siblings are more likely than controls to be diagnosed with AN, affective disorders, anxiety disorders, obsessive-compulsive disorder, and personality disorders
	Zhang et al. (2021) [94], Sweden	Diagnoses based on the ICD registered in nationwide databases	Siblings are more likely than general population to be diagnosed with schizophrenia, particularly brothers
General psychopathology	Adambegan et al. (2012) [82], Austria	Child Behavior Checklist (CBCL): Total problems	Prior to disease onset, general psychopathology was more prevalent in siblings who would later develop AN than in siblings who would remain healthy
	Amianto et al. (2011) [83], Italy	Symptom Checklist-90 (SCL-90): General Psychopathology	Siblings are more similar to controls than to their AN-affected siblings in general psychopathology
	Halvorsen et al. (2005) [86], Norway	Young Adult Self-Report (YASR) or equivalent Youth Self-Report (YSR) for underage subjects: Total problems Young Adult Behavior Checklist (YABCL): same as YASR/YSR but reported by parents/caregivers	Siblings of former AN probands score lower on general psychopathology, both in self- and in parental reports. Correlations between mothers’ and fathers’ reports were lower for siblings than for former AN probands. Correlations between self- and parental reports were high for former AN probands and low for siblings
	van Langenberg et al. (2016) [93], Australia	Strengths and Difficulties Questionnaire (SDQ): Total difficulties	Siblings self-report significantly more difficulties than children from the general population both before and after taking part in their affected sibling’s familial therapy. Mothers and fathers—and particularly fathers before familial therapy—seem to underreport siblings’ difficulties compared to the sibling’s self-reports, and assimilate them to the children from the general population
Internalizing psychopathology	Adambegan et al. (2012) [82], Austria	Child Behavior Checklist (CBCL), Internalizing behavior: Withdrawn behavior, Somatic complaints, Anxiety and depression, Social problems, Thought problems, Attention problems	Prior to disease onset, internalizing psychopathology was more prevalent in siblings who would later develop AN than in siblings who would remain healthy, with the largest size effects – particularly for anxious/depressed mood and social withdrawal
	Amianto et al. (2011) [83], Italy	Symptom Checklist-90 (SCL-90): Somatization, Obsessive-Compulsivity, Relational Sensitivity, Depression, Anxiety, Phobic Anxiety, Paranoid Ideation	Siblings are more similar to controls than to their AN-affected siblings in internalizing psychopathology except for Obsessive-Compulsivity, where siblings score higher than controls
	Halvorsen et al. (2005) [86], Norway	Young Adult Self-Report (YASR) or equivalent Youth Self-Report (YSR) for underage subjects, Internalizing scale: Anxious/Depressed and Withdrawn syndrome scales Young Adult Behavior Checklist (YABCL): same as YASR/YSR but reported by parents/caregivers	Siblings of former AN probands score lower on internalizing psychopathology, both in self- and in parental reports, particularly on the Anxious/Depressed syndrome scale
	Matthews et al. (2021) [89], United States of America	Child Behavior Checklist (CBCL), Internalizing behavior: Withdrawn behavior, Somatic complaints, Anxiety and depression, Social problems, Thought problems, Attention problems	Siblings are similar to controls in terms of internalizing psychopathology Hospitalization of the AN proband significantly increases emotional, hyperactivity/inattention and total symptoms experienced by siblings
	van Langenberg et al. (2016) [93], Australia	Strengths and Difficulties Questionnaire (SDQ): Emotional difficulties	Siblings self-report significantly more emotional difficulties than the general population both before and after participating in familial therapy with AN probands. Their mothers only report them suffering from more emotional symptoms than the general population after the family has finished therapy. Their fathers do not report them having more emotional difficulties than children from the general population at any point Emotional difficulties significantly correlate with duration of illness in AN probands
Externalizing psychopathology	Adambegan et al. (2012) [82], Austria	Child Behavior Checklist (CBCL), Externalizing behavior: Aggressive behavior, Delinquent behavior	Prior to disease onset, externalizing psychopathology was more prevalent in siblings who would later develop AN than in siblings who would remain healthy, though to a lesser extent than internalizing and general psychopathology
	Amianto et al. (2011) [83], Italy	Symptom Checklist-90 (SCL-90): Hostility, Psychoticism	Siblings are more similar to controls than to their AN probands in externalizing psychopathology
	Halvorsen et al. (2005) [86], Norway	Young Adult Self-Report (YASR) or equivalent Youth Self-Report (YSR) for underage subjects, Externalizing scale: Intrusive, Aggressive and Delinquent behavior syndrome scales + Mean Substance Use scale Young Adult Behavior Checklist (YABCL): same as YASR/YSR but reported by parents/caregivers	Both former AN probands and siblings score low on externalizing psychopathology compared to normative scores, but parental reports of former AN probands are higher compared to self-reports High and significant correlations between externalizing and internalizing symptoms are found in the reports from all informants with the only exception of fathers’ reports of healthy siblings
	Matthews et al. (2021) [89], United States of America	Child Behavior Checklist (CBCL), Externalizing behavior: Aggressive behavior, Delinquent behavior	Siblings are similar to controls in terms of externalizing psychopathology
	van Langenberg et al. (2016) [93], Australia	Strengths and Difficulties Questionnaire (SDQ): Conduct problems, Hyperactivity/Inattention, Peer problems, Prosocial behaviors	Siblings score similarly to the general population in conduct problems and both fathers and mothers seem to underreport these problems in siblings before familial therapy. Siblings report more hyperactivity/inattention symptoms than the general population after familial therapy. Both mothers and fathers tend to underreport these symptoms Siblings report more peer problems than the general population after familial therapy. Both mothers and fathers consider siblings’ peer problems to be comparable to general population Siblings report prosocial behaviors similar to the general population. Mothers tend to report less prosocial behaviors in siblings compared to normative data both before and after familial therapy, while fathers do so only after familial therapy
Negative mood (depressive and anxiety symptoms)	Amianto et al. (2011) [83], Italy	Beck Depression Inventory (BDI)	Siblings and controls score similarly and lower than AN probands
	Matthews et al. (2021) [89], United States of America	Children’s Depression Inventory-2 Short Version (CDI-2S) Multidimensional Anxiety Scale for Children-Second Edition (MASC-2)	Siblings score significantly higher than controls on anxiety symptoms and also on depressive symptoms at trend level
	Phillipou et al. (2022) [90], Australia	Depression Anxiety Stress Scale (DASS-42): Depression, Anxiety, Stress	Siblings did not differ from weight-restored AN probands nor healthy controls in depressive symptoms, but siblings and AN probands (both current and weight-restored) scored higher on anxiety compared to healthy controls
Obsessive-compulsive symptoms or traits Perfectionism	Amianto et al. (2011) [83], Italy	Symptom Checklist-90 (SCL-90): Obsessive-Compulsivity	Siblings present more obsessive-compulsive symptoms than controls but less than AN probands, both quantitatively (number of symptoms) and qualitatively (degree of functional impairment)
	Degortes et al. (2014) [84], Italy	ESTATE Lifetime Diagnostic Interview, part 2, for measurement of childhood and adolescent obsessive-compulsive traits: Perfectionism, Inflexibility, Rule-bound trait, Drive for order and symmetry, Excessive doubt and cautiousness	Siblings score similarly to controls on all subdomains considered. AN probands score higher than siblings and controls on the overall perfectionism, inflexibility, and doubt/cautiousness subdomains Obsessive-compulsive traits in childhood and adolescence increase risk of AN and also severity of AN once it is diagnosed in terms of more disordered eating and more general psychopathology
	Phillipou et al. (2022) [90], Australia	Multidimensional Perfectionism Scale (MPS): Concern over mistakes, Personal Standards, Parental expectations, Parental criticisms, Doubts and actions, Organisation, Overall	Siblings score similarly to controls on Overall perfectionism, concern over mistakes and personal standards, but both siblings and AN probands score higher than controls in parental criticisms (shared environment)
	Thornton et al. (2017) [92], United States of America	Multidimensional Perfectionism Scale (MPS): Concern over mistakes, Personal standards, Doubts about actions	AN probands are more perfectionist than twin siblings as measured with this group of subscales
Disordered eating	Amianto et al. (2011) [83], Italy	Eating Disorders Inventory (EDI-2) Binge-Eating Scale (BES)	Siblings are more similar to controls than to AN probands, with the exception that siblings tend to score lower than controls in Bulimia and higher in Body dissatisfaction and Inadequacy Siblings score lower than controls in binge-eating
	Areemit et al. (2010) [76], Canada	Eating Attitude Test-26 (EAT-26)	Siblings as a group show heterogeneous scores. Sisters score higher than brothers
	Dimitropoulos et al. (2013) [85], Canada	Eating Disorder Examination Questionnaire (EDE-Q)	Siblings score consistently lower than AN probands
	Karwautz et al. (2001) [88], United Kingdom	Eating Disorders Inventory-2 (EDI-2)	Siblings score consistently lower than AN probands
	Maloney & Shepard-Spiro (1983) [64], United States of America	Eating Attitude Test (EAT): Dieting, Bulimia and Food preoccupation, Oral control	Siblings and controls score similarly and lower than AN probands. However, 9.5% of the sibling sample score within the anorectic range on Dieting, finding later confirmed through clinical interview
	Phillipou et al. (2022) [90], Australia	Eating Disorder Examination Questionnaire (EDE-Q): Restraint, Eating concern, Shape concern, Weight concern, Global	Siblings do not differ from healthy controls on any of the EDE-Q subscales considered
Body image distortion or discomfort	Amianto et al. (2011) [83], Italy	Body Shape Questionnaire (BSQ)	Siblings are more similar to controls than to AN probands in terms of body shape concerns
	Benninghoven et al. (2008) [66], Germany	Perceptual body size distortion and self-ideal discrepancy (computed as the difference between objective, desired and perceived measurements using a somatomorphic matrix) Body image questionnaire (FKB-20)	Sisters and female controls obtain similar results. Brothers tend to overestimate their proportion of body fat compared with male controls, who slightly underestimated it. Neither brothers nor sisters seem to have more body image problems than controls in this study

## Data Availability

The data presented in this study are available on request from the corresponding author if intended to be used for justified academic and scientific purposes.

## Supplementary Materials

The following supporting information can be downloaded at: https://www.mdpi.com/article/10.3390/jcm15051772/s1, PRISMA 2020 Checklist.

## Abbreviations

The following abbreviations are used in this manuscript:

AN	Anorexia Nervosa
ED	Eating Disorder
ANr	Anorexia Nervosa, restricting subtype
ANp	Anorexia Nervosa, binging/purging subtype
OSFED-AN	Otherwise Specified Feeding and Eating Disorder, type Atypical Anorexia Nervosa
c-AN	Current Anorexia Nervosa
wr-AN	Weight-restored Anorexia Nervosa
BN	Bulimia Nervosa
